# The Relationship Between Mental Health Literacy and Social Well-Being: A Longitudinal Study in China

**DOI:** 10.3390/bs15010029

**Published:** 2024-12-30

**Authors:** Jiali Pan, Tianyu Xu, Dan Li

**Affiliations:** 1 Mental Health Education and Counseling Center, Shanghai Business School, Shanghai 201400, China; panjl@sbs.edu.cn; 2 School of Psychology, Shanghai Normal University, Shanghai 200234, China; xuty_psy@163.com

**Keywords:** mental health literacy, social well-being, late adolescent, cross-lagged model, latent change score model

## Abstract

In this study, 793 college students were examined through the utilization of the socioeconomic status scale, mental health literacy scale, and social well-being questionnaire at T1 and T2, respectively, with the aim of exploring the relationship between mental health literacy and social well-being and the relative static and dynamic development of the two. The results indicated that mental health literacy was significantly and positively correlated with social well-being to a moderate extent (T1: *r* = 0.31; T2: *r* = 0.35). Furthermore, the across-lagged model was employed to determine the relationship between mental health literacy and social well-being over time, revealing that mental health literacy and social well-being merely have a unidirectional predictive relationship; social well-being at T1 can significantly and positively predict mental health literacy at T2, but mental health literacy at T1 cannot predict social well-being at T2. We carried out the latent change score model and discovered that a higher level of T1 social well-being can facilitate the enhancement of mental health literacy subsequently.

## 1. Introduction

Mental health literacy (MHL) is a multifaceted and evolving concept characterized by various definitions (Jorm, 2015; Jorm et al., 1997; Wei et al., 2015). Jorm and his colleagues, who were the pioneers in introducing the notion of MHL, defined it as “knowledge and beliefs about mental disorders which aid their recognition, management or prevention” (Jorm et al., 1997). For an extended period, researchers have mainly concentrated on the prevention and intervention of mental disorders rather than on promoting well-being and personal development (Jorm, 2000; Jorm, 2012). There is an increasing consensus among scholars that mental health literacy encompasses individuals’ knowledge, beliefs, and behaviors related to mental disorders, including an understanding of how to achieve and maintain positive mental health, a comprehensive awareness of mental disorders and their treatments, efforts to reduce the stigma associated with these conditions, as well as strategies for enhancing self-help and supporting others (Bjornsen et al., 2017; Kutcher et al., 2015, 2016). Most of the existing MHL measures adopt self-reported evaluations of a single dimension, such as a person’s beliefs or knowledge regarding depression (Gabriel & Violato, 2010; Wang et al., 2013). Spiker and Hammer (2019) contend that MHL ought to be multiconstruct rather than single-dimensional. This contention is upheld by Jiang’s team, which not only incorporates mental illness and health promotion but also considers two dimensions of self-seeking help and assisting others, and can enhance individuals’ mental health literacy from three aspects: knowledge, attitude, and behavior (Jiang et al., 2020).

As a critical component of health literacy, it is essential for enhancing both individual and population health outcomes (Kajawu et al., 2016; Zhong et al., 2024). Previous investigations have demonstrated that low mental health literacy is correlated with adverse mental health conditions, such as depression and anxiety (Jacka et al., 2017; Tambling et al., 2023), stress (Tambling et al., 2023), internalized stigma (Tambling et al., 2023), suboptimal sleep quality (Jacka et al., 2017), and unhealthy lifestyles (Tambling et al., 2023). However, there are also studies indicating that individuals diagnosed with specific mental health disorders exhibit higher levels of MHL (BinDhim et al., 2023; O’Connor & Casey, 2015; Zeng et al., 2023). This might imply that people with mental illnesses are more prone to seek information on the subject, which promotes positive health behaviors and subsequently leads to enhancements in MHL. Brijnath et al. (2016) carried out a meta-analysis of 14 intervention investigations concerning MHL from 2000 to 2015 and discovered that enhancing MHL ameliorates mental health outcomes. For major mental illnesses, it is widely acknowledged that increasing public awareness of preventive measures, early intervention strategies, and treatment options can significantly benefit individuals (Jafari et al., 2021; Ming & Chen, 2020). Elevated levels of mental health literacy empower individuals to recognize mental illness at an early stage, reduce the stigma surrounding mental disorders, and facilitate access to timely and effective support and treatment, thereby enhancing their overall mental health and quality of life (Jafari et al., 2021; Ming & Chen, 2020; Morales-Torres et al., 2023; Tang et al., 2024). It is evident that mental health literacy is closely associated with mental health.

As one of the indicators closely related to mental health, well-being has garnered the attention of numerous researchers. Concurrent with the progress of well-being theory and empirical research, the conceptual orientation of well-being manifests two principal viewpoints. The prevailing perspective on hedonism suggests that well-being encompasses the pursuit of pleasure or the acknowledgment of distressing encounters (Kahneman et al., 1999); eudaimonism reflects the perspective that well-being encompasses not only being happy but also realizing human potential (Waterman, 1993). Thus, from the eudaimonic perspective, subjective well-being cannot be equated with well-being (Diener, 2009; Ryan & Deci, 2001). Prior research on well-being has predominantly concentrated on individual-level factors, such as subjective well-being and physical health, while neglecting social-level determinants, including social adjustment and social support (Larson, 1993). Mental well-being is increasingly regarded as comprising happiness, contentment, subjective well-being, self-realization, and positive functioning (Ryan & Deci, 2001). In fact, well-being is not merely manifested in terms of satisfaction with relationships, family life, employment, health, and finances, but also in terms of the connections with various aspects of the environment (Moser, 2009). In 1947, the World Health Organization established a conceptual link between mental health and social health, and defined health as a state of complete physical, mental, and social well-being. Since then, a growing body of research has focused on social well-being, with most scholars believing that social health and social well-being are synonymous (Diener, 2009). Keyes (1998) defined social well-being as individuals’ appraisal of their present social circumstances and their social functions, which reflects the significance or value of the realization of personal skills for others or society, as well as whether social skills are in a propitious state. According to Keyes, social well-being includes five aspects: social integration, social actualization, social contribution, social acceptance, and social coherence (Keyes, 1998). Keyes further maintains that well-being represents a comprehensive state of individual psychological experience and the integration of emotional well-being, psychological well-being, and social well-being, which are interrelated yet independent (Keyes, 2005, 2007; Ryff & Keyes, 1995). These three types of indicators of mental health, respectively, mirror the individual’s evaluation of their own quality of life in three aspects, namely, their sense of a good life, proper self-functioning, and sound social functioning (Keyes, 2003, 2007). Through confirmatory factor analysis, it is revealed that the goodness of fit of the three-factor model of emotional well-being, psychological well-being, and social well-being is significantly higher than that of the single-factor and two-factor models (Gallagher et al., 2009). To sum up, well-being encompasses three dimensions that possess certain correlation and independence: emotional well-being, psychological well-being, and social well-being. A considerable number of studies contend that positive mental health literacy is positively associated with subjective well-being, and positive mental health literacy can positively predict subjective well-being (Bjornsen et al., 2019; Mahmoodi et al., 2023; Nalipay et al., 2024). Previous research has mostly approached the relationship between mental health literacy and well-being from an individual perspective. However, as members of social groups, individuals are influenced by interactions with others and the social environment, which in turn affects their self-assessment of their environment and social functions. This well-being, which places emphasis on sociality, is equally important for individual development. However, limited knowledge is available regarding the associations between mental health literacy and social well-being (de Carvalho et al., 2022). The relationship between mental health literacy and social well-being is still unclear.

Some researchers assert at the theoretical level that mental health literacy could be one of the significant related factors influencing social well-being. Firstly, the ecological systems theory elucidates the promotional impact of mental health literacy on social well-being (Bronfenbrenner, 1979). This theory highlights the dynamic interaction between individuals and the environment, and posits that people’s psychology and behavior are the outcomes of the interaction with their environmental system. This environmental system encompasses multiple levels, ranging from the micro individual environment to the macro social and cultural environment, which jointly affect individual psychological development and well-being. Individuals with higher mental health literacy are capable of coping more effectively with challenges and pressures in life (Winding et al., 2023), maintaining a positive attitude and emotional state, making better utilization of social support resources, establishing positive interpersonal relationships, enhancing their social competence, and having positive social ties, thereby augmenting social well-being. Secondly, the theory of positive psychology (Seligman & Csikszentmihalyi, 2000) also discloses the facilitating effect of mental health literacy on social well-being. This theory emphasizes the significance of positive emotions and coping styles for individual mental health and well-being. Individuals with high mental health literacy are better equipped to identify, express, and regulate their emotions, and adopt positive behavioral strategies, such as seeking social support and participating in community activities (Cicognani et al., 2008; Son & Wilson, 2012). Individuals experience more positive emotions in daily life, which enhances the connections and activities between individuals and others and the community, thus enhancing social well-being. Based on the foregoing theories, we hypothesize that mental health literacy exerts a positive predictive effect on social well-being.

On the other hand, social well-being may also be predictive of mental health literacy. The cognitive behavioral theory contends that an individual’s emotions, behaviors, and responses are shaped by their cognitive processes, such as thinking, beliefs, and assumptions. When individuals experience a higher level of social well-being, they are more inclined to hold positive cognitive appraisals of the social environment (e.g., the society is improving, I have made my own contribution to the development of the society, people are willing to assist others, etc.), establish healthier thinking patterns and behavioral habits, and thereby fulfill interpersonal functions optimistically and confidently (Baldwin et al., 2020). This positive cognitive and behavioral pattern contributes to further enhancing individuals’ mental health literacy and makes them more disposed to seek help when they encounter mental health problems. Similarly, the ecological systems theory and the theory of positive psychology also uphold this perspective, believing that the well-being individuals experience in the social environment will have a positive influence on their mental health literacy. A social environment replete with support, understanding, and acceptance can assist individuals in forming positive self-cognition and self-efficacy, thereby improving their mental health literacy level. Based on the above reason, we propose a hypothesis that social well-being has a positive predictive effect on mental health literacy.

In current study, firstly, we used an integrated multidimensional concept of mental health literacy, including self–others, mental health–mental disorders, and knowledge–attitude–behavior, to explore the correlation between mental health literacy and social well-being in the Chinese college students’ group. Secondly, cross-lagged analysis was used to explore the prediction direction of mental health literacy and social well-being from the static level. Lastly, a latent change score model was used to further verify the relationship between the two variables, and the interactive relationship between the rate of change of mental health literacy and social well-being was explored at the dynamic level. This study aimed to deepen our comprehension of the relationship between mental health literacy and social well-being, illuminating the potential effects and implications of mental health literacy.

## 2. Method

### 2.1. Participants

The G*power software (Version 3.1.9.7) was utilized in this study to conduct a power analysis and calculate the necessary sample size. We aimed for a power of 0.95 and set the α level at 0.05 with an expected effect size of 0.15 (Faul et al., 2007). G*Power eventually determined a total sample size of 107 participants, which provided acceptable statistical power. The final sample size of the study was determined after accounting for potential attrition or missing data during data collection.

The study was carried out with 795 participants from a business college in Shanghai, China, at the T1 time point, which was in the early fall semester. The second test was conducted four months later. Both tests were executed in the form of coursework, and students received corresponding credits. However, due to illness, absence, etc., two participants did not participate in the T2 time point test. After excluding these participants without T2 data, 793 participants were included in the study. The age range of participants at T1 time point was 17–31 (*M* = 19.23, *SD* = 0.76). Among these participants, there were 507 female (63.9%), 285 male; 448 participants were single children (43.5%), 345 participants were non-single children; 621 participants were from urban areas (78.3%), 172 participants were from rural areas.

### 2.2. Measures

#### 2.2.1. Social Economic Status

The Social Economic Status Scale (SES) included three indicators of parental occupation type, parental education level, and income, with a total of 5 items (Jiang et al., 2021). Among these, the occupation type was divided into 11 categories, the education level into 4 categories, and the income into 6 categories based on the results of the 2022 China’s National Economic and Social Development Statistical Bulletin. In this study, SES score was obtained by converting three indicators of parental occupation type, parental education level, and income into rank variables and using factor analysis to carry out weighted transformation. The specific approach was as follows: Firstly, the average values of parental occupation type and parental education level were calculated, respectively. Then, the 10 levels of occupation type (excluding school students) were assigned scores of 1–10 in ascending order of points from low to high. The 4 levels of education were assigned 1–4 points from in the same manner, and the 6 levels of per capita disposable annual income of families were assigned 1–6 points from low to high. Then, the three indexes were converted into standard scores, and principal component analysis was carried out to obtain a principal factor with characteristic root greater than 1, which explained 66.29% of the variance. Finally, using the load coefficients of the three indexes on the principal factors, the calculation formula of SES was made as follows: SES = (0.856 × Z_occupation type_ + 0.843 × Z_education level_ + 0.738 × Z_income_)/1.989. Among them, 0.856, 0.843, and 0.738 were the factor loads of the three factor indexes, respectively, and 1.989 was the characteristic root of the first factor. The higher the SES score, the higher the socioeconomic status.

#### 2.2.2. Mental Health Literacy

Mental health literacy was measured using the Mental Health Literacy Questionnaire (MHLQ) developed by Wu et al. (Wu et al., 2023). This 60-item scale consists of six dimensions: mental illness knowledge (e.g., “People who have good relationships with others do not have mental illness”), mental health knowledge (e.g., “One’s mental health state is stable and unchanging”), attitudes towards mental illness (e.g., “Mental illness is a minor problem, and we don’t need pay too much attention to it”), attitudes towards mental health (e.g., “I think my mental health is the most important”), behaviors to cope with mental illness (e.g., “When I feel down or lack energy and this doesn’t get better for some time, I’ll seek professional help”), and behaviors to maintain mental health (e.g., “I know how to find information and knowledge about mental health”). Thirty true/false/don’t know items assessed MHL knowledge, of which “True” responses were coded as 1 and other responses were coded as 0. It was scored using a 5-point Likert scale (1 = strongly disagree, 5 = strongly agree) to assess MHL attitude and behavior—the two options in the same correct direction as the score were coded as 1, and the other options were coded as 0. The MHL score is the sum of the values of all items. A higher total score indicates a greater level of mental health literacy. The Cronbach’s α coefficient of the scale in this study at two time points were 0.85 and 0.91, respectively.

#### 2.2.3. Social Well-Being

The Social Well-being Scale was employed to measure the participants’ level of social well-being (Miao & Wang, 2009), which was revised by Miao and Wang to be suitable for Chinese (Keyes & Shapiro, 2003). This scale comprises 15 items and consists of five dimensions (e.g., “The community I belong to makes me feel good”), including social acceptance, social actualization, social contribution, social coherence, and social integration. The 5-point Likert scoring method was adopted in this scale, and the total score is calculated by summing all items. The higher total score indicates the higher level of social well-being. The Cronbach’s α coefficients at two time points in current study were 0.89 and 0.93, respectively.

### 2.3. Statistical Analyses

The data were statistically analyzed by SPSS 20.0, Mplus 8.3, and R 4.4.1 software. Firstly, SPSS 20.0 was used for data collection, descriptive statistical analysis, correlation analysis, and the common method deviation test. Secondly, Mplus 8.3 was used to construct a cross-lagged model to test the model fit, exploring how a variable affects itself and other variables over time. Finally, we further investigated the relationship between the rate of change among variables and built a bivariate latent change score model through R 4.4.1 software to explore the impact of the initial state of a variable on the subsequent development rate of itself or other variables at a dynamic level (Kievit et al., 2018).

## 3. Result

### 3.1. The Common Method Deviation Test

The data in this study were based on self-reported questionnaires, which may lead to common method bias. In the process of testing, the anonymity and confidentiality of data were emphasized to control the common source of method deviation. In addition, the result of Harman’s single-factor test showed that there were 23 factors at T1 and 17 factors at T2 with eigenvalues greater than 1, respectively. The total variance explained by the first factor was 13.08% and 18.75%, which was below the critical value of 40% (Podsakoff et al., 2003), indicating that there was no severe common method bias in this study.

### 3.2. Descriptive and Correlation Analysis

The results of descriptive statistics and correlation analysis are shown in Table 1. The mean scores of mental health literacy and social well-being across the two time points (T1 and T2) ranged from 39.11 to 40.60 and from 51.61 to 54.61, respectively. Correlation analysis of the measured variables in the two time points (T1 and T2) manifested that T1 was significantly positively correlated with T2 both in mental health literacy (*r* = 0.58, *p* < 0.05) and in social well-being (*r* = 0.64, *p* < 0.05). At both time points, mental health literacy and social well-being were significantly positively correlated with each other (*r* = 0.31, *p* < 0.05; *r* = 0.35, *p* < 0.05). Mental health literacy at two time points was significantly negatively associated with gender (T1: *r* = −0.17, *p* < 0.05; T2: *r* = −0.23, *p* < 0.05), and significantly positively associated with single children (T1: *r* = 0.08, *p* < 0.05; T2: *r* = 0.08, *p* < 0.05) and SES (T1: *r* = 0.08, *p* < 0.05; T2: *r* = 0.08, *p* < 0.05). In addition, at any point in time, social well-being was not significantly related to gender, single children, or SES.

### 3.3. Longitudinal Cross-Lagged Model Analysis

**Figure 1:**
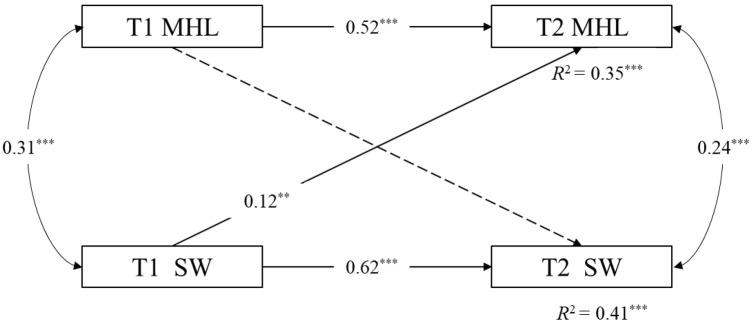
Cross-lagged model. Note. ** *p* < 0.01, *** *p* < 0.001. MHL = mental health literacy. SW = social well-being. Nonsignificant paths are represented by dotted lines. The paths from the covariates gender, single children, and SES are not presented for reasons of clarity. All path coefficients are standardized.

As shown in Figure 1, a cross-lagged model was used to determine the relationship between mental health literacy and social well-being over time. Controlling for gender, single children, and SES, the model fit of the cross-lagged model had an acceptable fit to the data (χ^2^/df = 38.31/6, CFI = 0.94, TLI = 0.89, RMSEA = 0.08, SRMR = 0.05). Cross-lagged paths indicated that all variables at T1 could predict themselves at T2. To be specific, the level of initial mental health literacy could significantly predict the level of subsequent mental health literacy (*β* = 0.52, *p* < 0.05), and the level of initial social well-being could significantly predict the level of subsequent social well-being (*β* = 0.62, *p* < 0.05). In addition, the results indicated that one variable at T1 can predict another variable at T2. Social well-being at T1 could significantly and positively predict mental health literacy at T2 (*β* = 0.12, *p* < 0.05). However, mental health literacy at T1 could not significantly predict social well-being at T2 (*β* = 0.05, *p* > 0.05).

### 3.4. Latent Change Score Model Analysis

**Figure 2:**
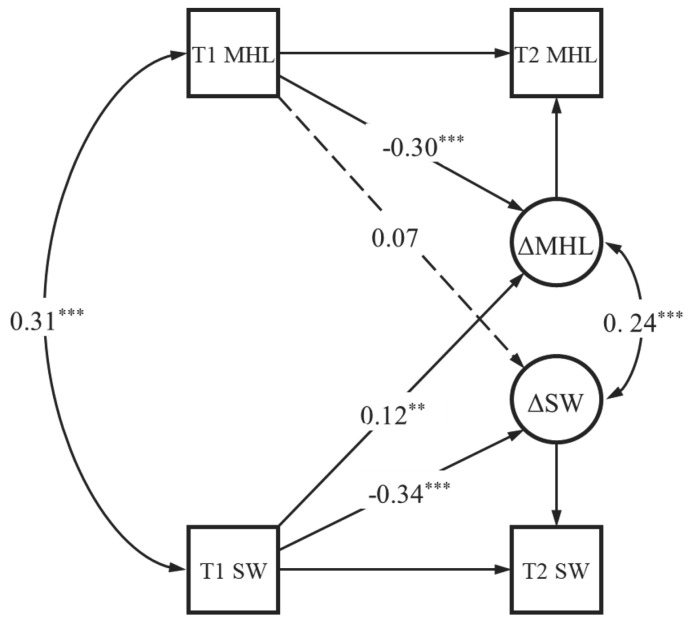
Latent change score model. Note. ** *p* < 0.01, *** *p* < 0.001. MHL = mental health literacy. SW = social well-being. All path coefficients of solid line are standardized, except for unlabeled paths that are constrained to 1. Nonsignificant paths are represented by dotted lines.

The latent change score model of the relationship between mental health literacy and social well-being is presented in Figure 2. The data obtained a saturated model. The results of self-feedback effect demonstrated that prior levels of mental health literacy could significantly and negatively predict subsequent changes in mental health literacy (*β* = −0.30, *p* < 0.05), indicating an overall slowing of growth over time. That is, compared with individuals with a high level of mental health literacy, individuals with a low level of mental health literacy would increase their mental health literacy ability faster in the future. A similar pattern was found for social well-being (*β* = −0.34, *p* < 0.05). The results of the cross-domain coupling effect demonstrated that prior levels of social well-being could significantly and positively predict subsequent changes in mental health literacy (*γ* = 0.12, *p* < 0.05), indicating that higher levels of social well-being in the previous time point (T1) could facilitate the rate of growth change in mental health literacy. However, the prior levels of mental health literacy could not predict subsequent changes in social well-being (*γ* = 0.07, *p* > 0.05). The above results showed that social well-being is a leading variable in the developmental process.

## 4. Discussion

The aim of this study was to investigate the relationship between mental health literacy and social well-being in college students (i.e., early adulthood) and to examine the predicted directivity of relative static and dynamic development changes between the two.

The social well-being and mental health literacy of young adults in China were evaluated in this study, and both scores were higher than half of the total, demonstrating that they are at medium to high levels. Our results found that there was a significant positive correlation between social economic status and mental health literacy; that is, individuals with higher socioeconomic status have higher levels of mental health literacy, which is consistent with the findings of Jiang et al. (Jiang et al., 2021). As China’s social and economic development proceeds, people’s priorities move from basic material requirements to psychological health demands, resulting in an increased focus on mental health. As a result, as social and economic conditions have improved, so too has mental health literacy. When controlling for age and gender, research shows that socioeconomic disadvantage is strongly related with depression symptoms in early adulthood, implying that higher income levels predict fewer depressed symptoms (Ferschmann et al., 2024). This corresponds to our research findings. It is clear that social and economic development levels can influence individuals’ motivations for psychological health needs, hence improving mental health. Meanwhile, the advancement of many parts of society fosters individuals’ social relationships and improves social well-being.

In addition, the results of this study show that gender is significantly related to mental health literacy, as shown by females having higher levels of mental health literacy. This result further supports the view of previous studies (Holzinger et al., 2012; Jiang et al., 2021; Tay et al., 2018). A systematic review found gender differences in mental health literacy (Holzinger et al., 2012). On the one hand, for themselves, females were more likely to seek informal and formal help (family, doctors, psychological counselors, etc.) (Furnham et al., 2014) and had a higher propensity to seek help (Tay et al., 2018). On the other hand, females were more likely to recommend professional help for other people (Coles et al., 2016; Holzinger et al., 2012). Mental health literacy is not only the individual’s understanding of mental health knowledge, but also includes the knowledge learning, attitude internalization, and behavior performance of their own and others’ mental health or disease (Jiang et al., 2021; Wu et al., 2023). Compared with males, females tended to be more accommodating, more helpful, more agreeable, and had more prosocial behavior (Holzinger et al., 2012; Kuhnert et al., 2017). These characteristics make females, while focusing on themselves, often hopeful of improving their mental health literacy by increasing their knowledge of mental health and disease prevention (knowledge level), having a greater willingness to help people around them (such as family, friends, and even strangers) (attitude level), and then adopting corresponding behavioral strategies conducive to mental health (behavior level).

The results in this study indicated that single children had higher levels of mental health literacy. Although there were few studies exploring the mental health literacy of single children, numerous studies have shown that single children are able to receive more family attention (Chen & Zhou, 2019; Liu & Jiang, 2021). Due to the influence of traditional Chinese culture, the single children in China are often favored by parents and grandparents, and have more educational resources, family support, and material foundation. A study of junior high school students in China found that single children tended to have closer relationships with their parents than children from multiple-child families (Liu & Jiang, 2021), and that parents were more concerned about their children’s physical and mental health (Chen & Zhou, 2019). This positive family relationship can effectively promote parent–child communication and imperceptibly pass on parents’ knowledge, attitude, and behavior of mental health to their children.

Is mental health literacy related to social well-being? The results of this study found that mental health literacy was significantly positively correlated with social well-being to a moderate degree (T1: *r* = 0.31; T2: *r* = 0.35), which is consistent with previous studies (Bjornsen et al., 2019; de Carvalho et al., 2022; Gorczynski et al., 2020; Mahmoodi et al., 2023; Nalipay et al., 2024; Zhang et al., 2023). A study of young and middle-aged people found that individuals with a high level of mental health literacy tended to have higher levels of well-being (Zhang et al., 2023). Similar results were found in the study of 15–21-year-old adolescents, with a significantly positive correlation between mental health literacy and well-being (Bjornsen et al., 2019). It is worth noting that in previous studies, there was only a weak positive correlation between mental health literacy and well-being (Bjornsen et al., 2019; Mahmoodi et al., 2023; Nalipay et al., 2024), or even no significant correlation (Hamzah et al., 2023). The reason may be that these researchers mostly used subjective well-being or psychological well-being (Gorczynski et al., 2020; Mahmoodi et al., 2023), which emphasize individual emotional experience and psychological function, respectively, and reflect the personal characteristics of well-being (Ye et al., 2023). As a social characteristic, social well-being refers to an individual’s self-assessment of the quality of their relationships with others, the collective, and society, as well as their living environment and social function (Keyes, 1998). As individuals, we are part of society and cannot function without social connection. Individuals are closely connected with themselves, others, and their environment, and they evaluate their quality of life and personal functioning based on social standards. Mental health literacy refers to the knowledge, attitudes, and behavioral habits developed to promote mental health and cope with mental disorders. It encompasses not only aspects related to oneself but also those related to others (Jiang et al., 2021; Wu et al., 2023). This means that an individual’s mental health literacy is not isolated; it is influenced by external factors and, in turn, affects the external environment or others, also possessing social characteristics. Therefore, compared with subjective well-being and psychological well-being, social well-being has a stronger correlation with mental health literacy. Our results confirm that social well-being, subjective well-being, and psychological well-being are distinct components of well-being, sharing commonalities as well as unique aspects (Gallagher et al., 2009). There are varying degrees of correlation between mental health literacy and different subtypes of well-being.

Mental health literacy and social well-being are merely a unidirectional predictive relationship. Our longitudinal cross-lagged study results found that social well-being at T1 can significantly positively predict mental health literacy at T2, but mental health literacy at T1 cannot predict social well-being at T2. This differs from previous cross-sectional study results (Bjornsen et al., 2019; Nalipay et al., 2024). Nalipay and other researchers explored the impact of positive mental health literacy on well-being, and they found that positive mental health literacy can positively predict well-being (Nalipay et al., 2024). Positive mental health literacy mainly focuses on how individuals can acquire and maintain mental health (Carvalho et al., 2022), while neglecting the aspect of coping with mental disorders. But, as Keyes’ dual-factor model of mental health suggests, mental health and mental disorder are not a one-dimensional, bipolar potential unity. Mental disorder and mental health do not lie at the ends of this continuum, respectively, but, rather, are two potential continuums (Keyes, 1998). The elimination of mental disorder does not mean the presence of mental health; neither the disease model nor the health model can independently describe the mental health of a population. Mental health and mental disorder are a combined assessment system. When the aspect of addressing mental disorder is integrated into mental health literacy, this excessive focus on mental disorder can trigger negative emotions such as anxiety and depression in individuals, thereby reducing their sense of well-being (Malone & Wachholtz, 2018). Previous studies have focused more on positive aspects of mental health literacy (Bjornsen et al., 2019; de Carvalho et al., 2022; Nalipay et al., 2024). When adopting this mental health literacy that integrates mental health with mental disorders, the effect of individual attention to mental health in promoting increased well-being is, thus, offset; therefore, mental health literacy cannot predict subsequent social well-being. In addition, highly sensitive personality traits may also affect the relationship between mental health literacy and social well-being. The Intense World Theory posits that some individuals have intense and excessive perceptions, attention, memory, and emotional responses to environmental information, and integration deficits further lead to various social withdrawal issues (Markram & Markram, 2010). Some studies have found that highly sensitive personalities often positively predict individuals’ internalizing problems (Burgard et al., 2022), and they are more likely to experience anxiety (Dosari et al., 2023). Compared to normal individuals, when these individuals are exposed to an environment with high mental health literacy, their own highly sensitive personality traits make them overly sensitive to mental disorders, resulting in more anxiety, depression, and other negative emotions. This, in turn, affects their social interactions, leading to social withdrawal, decreased satisfaction, and, ultimately, a decline in their sense of social well-being. The diversity of these personality traits may also be one of the reasons why mental health literacy cannot directly predict social well-being.

The cross-lagged model analysis showed results that were different from previous studies, finding that social well-being can predict subsequent mental health literacy. Keyes proposed in 1998 that social well-being has five dimensions, including social integration, social acceptance, social contribution, social actualization, and social coherence (Keyes, 1998). Social integration refers to individuals having a sense of belonging and being able to receive comfort and support from society. Healthy individuals feel like a part of society, and this sense of integration makes them feel a commonality with others who make up society. Therefore, individuals with a good sense of social well-being not only pay attention to their own mental health and mental disorders but also to the mental health and mental disorders of others, thereby having better mental health literacy. Individuals with high social well-being also tend to have stronger social acceptance. These individuals often perceive others as benevolent, have better relationships with others, possess higher levels of mental health literacy, and have a greater willingness to seek help (for themselves) and offer help (to others). Social contribution reflects an individual’s recognition of their social value, while also being acknowledged or valued by society and others. This recognition further promotes the development of the individual’s social actualization, making them believe that people, collectives, and society have the potential to develop and grow positively. This sense of self-efficacy regarding the positive aspects of society inspires individuals to strive to improve their own mental health literacy and also enhances their desire for society and others to have mental health as well. Thus, while maintaining one’s own mental health and addressing personal mental disorder, one should not forget to pay attention to these aspects in others and attempt to help them. Individuals with good social harmony are often interested in life, can find meaning in social life, and have a certain degree of understanding. We experience countless life events every day, some positive and some negative, some predictable and some unpredictable, some involving others and some involving ourselves. This requires individuals to coordinate various aspects well to achieve a degree of harmony (Miao & Zhao, 2009). The concept of mental health literacy aligns with this viewpoint, encompassing the maintenance of mental health (positive aspect) and the coping with mental disorder (negative aspect), self and others, knowledge, attitude, and behavior. Individuals with high social well-being can accept and pay attention to both the positive and negative aspects of things, respond actively to achieve harmony, and possess higher mental health literacy. It can be seen that social well-being can affect the positive development of individual mental health literacy.

The latent change score model reaffirms the conclusions of the cross-lagged model analysis from a dynamic change perspective. First, the higher the initial level of mental health literacy, the slower its subsequent rate of change. Social well-being exhibits the same developmental pattern. Second, only the initial level of social well-being can positively predict the subsequent changes in mental health literacy, meaning that a high level of social well-being can promote the rapid improvement of mental health literacy. Individuals with high social well-being have a better self-concept, self-esteem, and self-acceptance, are more satisfied with their personal lives, and hold a more optimistic attitude towards the future and society (Lluch-Canut et al., 2013). An individual’s positive inclination towards society, as well as their acceptance of others and different social characteristics, can encourage them to be willing to understand mental health knowledge, help others cope with mental disorder, and engage in corresponding positive behaviors. Therefore, individuals with high social well-being often experience a faster growth rate in their mental health literacy (Carvalho et al., 2022).

## 5. Limitations and Future Directions

First, the participants in this study were recruited from a business college in Shanghai, China, with a higher proportion of female students. Social culture, sampling range, and gender ratio may all affect the generalizability of the results. In the future, it is necessary to balance the gender ratio and expand the sampling range to include multiple regions, schools, and age groups to enhance the representability of the results and increase external validity. Furthermore, future research could compare the relationship between mental health literacy and social well-being across different cultural groups to explore whether the conclusions are cross-culturally consistent.

Second, this study only measured two time points, making it difficult to observe more subtle developmental changes in individuals’ mental health literacy and social well-being. Future research could further extend the interval and number of test points to explore the developmental changes in the relationship between mental health literacy and social well-being over time.

Third, although there is a unidirectional relationship between mental health literacy and social well-being, the mechanisms of their development are still unclear. Further exploration can be conducted on the roles of other variables in this relationship and the mediating mechanisms involved.

## 6. Conclusions

Our research expands on the relationship between mental health literacy and well-being, finding a moderate and significant positive correlation between mental health literacy and social well-being. Cross-lagged model analysis shows that mental health literacy at T1 can significantly and positively predict its own level at T2, and social well-being at T1 can similarly and significantly predict its own level at T2. Social well-being at T1 can positively predict mental health literacy at T2, meaning that higher social well-being is associated with higher mental health literacy. But mental health literacy at T1 cannot predict social well-being at T2. The latent change score model analysis shows that the self-feedback effect of mental health literacy is significant, meaning that the higher the initial level of mental health literacy, the smaller the subsequent development changes in mental health literacy. Social well-being exhibits the same pattern of self-feedback effect. Mental health literacy and social well-being only exhibit a unidirectional cross-domain coupling effect. Merely, the initial level of social well-being can positively predict the subsequent development changes in mental health literacy, implying that the higher the initial level of social well-being, the faster the growth rate of mental health literacy. This indicates that social well-being is the leading variable between the two.

## Figures and Tables

**Table 1:** Descriptive and correlation analysis of all variables.

	M ± SD	1	2	3	4	5	6	7
1. T1 MHL	39.11 ± 8.10	-						
2. T1 SW	51.61 ± 10.02	0.31 **	-					
3. T2 MHL	40.60 ± 10.15	0.58 **	0.27 **	-				
4. T2 SW	54.61 ± 11.25	0.25 **	0.64 **	0.35 **	-			
5. Gender	-	−0.17 **	0.02	−0.23 **	−0.01	-		
6. SC	-	0.08 *	−0.04	0.08 *	0.02	0.002	-	
7. SES	-	0.08 *	0.01	0.08 *	0.06	−0.06	0.43 **	-

Note. *N* = 793. * *p* < 0.05, ** *p* < 0.01. Gender is a dummy variable; female = 0, male = 1. SC = single children; SC is a dummy variable: non-single children = 0, single children = 1. SES = social economic status. MHL = mental health literacy. SW = social well-being.

## Data Availability

The data presented in this study are available on request from the corresponding author (the data are not publicly available due to privacy or ethical restrictions).
